# BLTR1 and CD36 Expressing Microvesicles in Atherosclerotic Patients and Healthy Individuals

**DOI:** 10.3389/fcvm.2018.00156

**Published:** 2018-10-30

**Authors:** Mathilde Sanden, Jaco Botha, Michael René Skjelbo Nielsen, Morten Hjuler Nielsen, Erik Berg Schmidt, Aase Handberg

**Affiliations:** ^1^Department of Clinical Biochemistry, Aalborg University Hospital, Aalborg, Denmark; ^2^Department of Clinical Medicine, Faculty of Medicine, Aalborg University, Aalborg, Denmark; ^3^Department of Cardiology, Aalborg University Hospital, Aalborg, Denmark

**Keywords:** atherosclerosis, extracellular vesicles, microvesicles, BLT1, flow cytometry, arachidonic acid, eicosapentaenoic acid, leukotriene b4 receptor

## Abstract

**Aims:** Monocytes/macrophages play a crucial role in the development, progression, and complication of atherosclerosis. In particular, foam cell formation driven by CD36 mediated internalization of oxLDL leads to activation of monocytes and subsequent release of microvesicles (MVs) derived from monocytes (MMVs). Further, pro-inflammatory leukotriene B4 (LTB4) derived from arachidonic acid promotes atherosclerosis through the high-affinity receptor BLTR1. Thus, we aimed to investigate the correlation between different MMV phenotypes (CD14^+^ MVs) on the one hand, and arachidonic acid and eicosapentaenoic acid contents in different compartments including atherosclerotic plaques, plasma, and granulocytes on the other.

**Methods and Results:** Samples from patients with femoral atherosclerosis and healthy controls were analyzed on an Apogee A60 Micro-PLUS flow cytometer. Platelet-poor plasma was labeled with lactadherin-FITC, anti-CD14-APC, anti-CD36-PE, and anti-BLTR1-AF700. Eicosapentaenoic acid and arachidonic acid content in different compartments in patients were analyzed using gas chromatography. Compared to controls, patients had lower levels of BLTR1^+^ MVs (*p* = 0.007), CD14^+^BLTR1^+^ MVs (*p* = 0.007), and CD14^+^BLTR1^+^CD36^+^ MVs (*p* = 0.001). Further, in patients CD14^+^ MVs and CD14^+^CD36^+^ MVs correlated inversely with arachidonic acid in granulocytes (*r* = −0.302, *p* = 0.039 and *r* = −0.322, *p* = 0.028, respectively). Moreover, CD14^+^CD36^+^ MVs correlated inversely with arachidonic acid in plasma phospholipids in patients (*r* = −0.315, *p* = 0.029), and positively with triglyceride in both patients (*r* = 0.33, *p* = 0.019) and controls (*r* = 0.46, *p* = 0.022).

**Conclusion:** This is the first study of its kind and thus the results are explorative and only indicative. BLTR1^+^ MVs and CD14^+^CD36^+^ MVs has potential as markers of atherosclerosis pathophysiology, but this needs further investigation.

## Introduction

Despite thorough investigation of atherosclerosis, the initial events that activate endothelial cells and circulating monocytes are still debatable. Atherosclerosis is characterized by chronic vascular inflammation, infiltration of lipid-laden macrophages, and blood vessel narrowing (1). However, it has been shown that pro-inflammatory mediators including TNF-α, IL-6, CRP, and other factors present in atherosclerotic conditions stimulate the formation of microvesicles (MVs). MVs are a heterogeneous population of cell-derived membrane-encapsulated particles ranging from 0.1 to 1.0 μm in size released by outward budding of the plasma membrane in latent, activated, stressed, and apoptotic cells. They are characterized by a phosphatidylserine (PS) rich outer membrane and by membrane proteins, receptors, and cytosolic materials including mRNA and microRNAs from their parental cell of origin giving an insight into the state of this cell (2). For that reason, a growing interest acknowledging the potential of MVs as biomarkers, in particular within diseases associated with vascular injury, inflammation and pro-thrombotic states has emerged and several studies have demonstrated an elevation of MVs originating from monocytes (MMVs) among others (3).

Another important player in the atherogenic process is the scavenger receptor and fatty acid transporter CD36 (4). Binding of oxidized low-density lipoprotein (oxLDL) and oxidized phospholipids (oxPL) by CD36 expressed on macrophages leads to internalization, foam cell formation and thickening of the vessel wall media and intima (5). Further, it has been suggested that polarization of pro-inflammatory macrophages underlying the low-grade inflammatory state in atherosclerosis is driven by CD36 exposure to oxLDL (6). In addition, binding of oxLDL to CD36 stimulates secretion of cytokines attracting immune cells that leads to further infiltration of intima and narrowing of the vessel (4). Moreover, circulating CD36 shows a general association with several cardio-metabolic conditions including atherosclerosis (7). Thus, enumerating the level of MMVs expressing CD36 may have the potential of detecting ongoing atherosclerosis.

The risk of atherosclerosis has also been associated with the intake of the marine n-3 polyunsaturated fatty acids (PUFAs), eicosapentaenoic acid (EPA) and docosahexaenoic acid (DHA), and the more abundant n-6 PUFAs, linoleic acid and arachidonic acid (AA) (8). Although pro-inflammatory leukotrienes (LTs) are synthesized from both EPA and AA through the 5-lipoxygenase pathway, LTs derived from EPA are much less potent than LTs derived from AA with the most potent being LTB4 (8). The pro-inflammatory signals are most likely promoted through the high-affinity receptor BLT1 (BLTR1) and the low-affinity receptor BLTR2 (9) and especially BLTR1 is suggested to be linked to the development and progression of atherosclerosis as studies have demonstrated that treatment with BLTR1 antagonists reduces atherosclerotic lesions in mice (10). Thus, we expected that the higher levels of LTB4 would increase the expression of BLTR1 and further activate BLTR1 expressing cells with higher levels of circulating BLTR1^+^ MMVs as a consequence. Besides increasing leukocyte/monocyte chemotaxis, binding of LTB4 to BLTR1 further enhances the expression of CD36 stimulating foam cell formation (11). Therefore, we hypothesized that levels of CD36^+^ and BLTR1^+^ MMVs are increased in atherosclerotic patients, and in particular in those with a high content of AA in granulocytes, plasma, and plaques. The aim of this study was to investigate the correlation between these MMV phenotypes on the one hand, and AA and EPA contents in different compartments including atherosclerotic plaques, plasma, and granulocytes on the other. To clarify this we compared levels of different circulating MMV phenotypes between participants with femoral atherosclerosis and healthy controls and further compared the MMV levels with the content of AA and EPA in the atherosclerotic participants. Finally, we investigated the associations between MMV levels and conventional risk factors of atherosclerosis. This might elucidate the potential of CD36 and BLTR1 bearing MMV phenotypes as novel biomarkers in predicting atherosclerosis.

## Methods

### Study population

This study included 48 participants (24 male/24 female) with symptomatic peripheral arterial disease undergoing femoral thrombendarterectomy as previously described (8) and 24 healthy controls (9 male/15 female, age >18 years). Exclusion criteria for the participants with femoral atherosclerosis were allergic asthma or rhinitis, systemic steroid treatment, chronic inflammatory diseases, acute coronary syndrome within the last 30 days, cancer, and inability to give informed consent. Exclusion criteria for the healthy participants were history of atherosclerotic disease, hypertension, and medication of any kind.

### Ethical aspects

Ethical approval of the study was granted by the Research Ethics Committee of the North Denmark Region (id. N-20100047). All participants gave written informed consent in accordance with the Declaration of Helsinki before inclusion.

### Analysis of leukotrienes and fatty acid composition in atherosclerotic plaques and plasma

Analyses of LTs and fatty acids in plaques have previously been described by Nielsen et al. (8). Briefly, for LTB4 quantification, plaques removed during femoral artery thrombendarterectomy were cut into smaller pieces and stimulated with calciumionophore A23187 followed by a solid phase immunoassay ELISA. Analyses of fatty acid composition in plaques and granulocytes were performed using gas chromatography as earlier described (8). The relative content of fatty acids including PUFAs in plasma phospholipids was analyzed by extraction of total lipids from plasma followed by separation and gas chromatography analysis (8). The intake of EPA and DHA was self-reported and only noted for the patients.

### Preparation and labeling of samples for MV analysis

Blood samples for flow cytometric analysis were collected into tubes containing sodium citrate anticoagulant at a 3.2% (0.105 M) final concentration and centrifuged within 1 h after collection. To obtain platelet-poor plasma (PPP) they were initially centrifuged at 1,800x g for 10 min at room temperature followed by centrifugation of supernatant at 3,000x g for 15 min and stored at −80°C until further analysis.

Upon analysis, freshly thawed PPP was vortexed shortly, centrifuged at 1,850x g for 5 min at room temperature followed by fluorescent labeling. Twenty-five micro liter centrifuged PPP was incubated for 30 min at 4°C in the dark with 2.5 μL fluorescein isothiocyanate- (FITC-) conjugated bovine lactadherin (83 μg/mL, Hematologic Technologies Inc., Vermont, USA), 2.5 μL allophycocyanin- (APC-) conjugated anti-human CD14 [200 μg/mL, IgG1, κ (clone 63D3, BioLegend, San Diego, CA, USA)], 2.5 μL phycoerythrin- (PE-) conjugated anti-human CD36 [200 μg/mL, IgG2α, κ, (clone 5-271, BioLegend, San Diego, CA, USA)], and 2.5 μL Alexa Fluor 700- (AF700-) conjugated anti-human BLTR1 [0.7 mg/mL, IgG2α, (clone 2020/7B1, Novus Biologicals, USA)]. A second incubation with matched isotype control antibodies were used as negative controls. After incubation, samples were diluted in 390 μl or 1,665 μl 0.22-μm sterile filtered PBS to reach a final dilution factor of 17 or 68.

### Analysis of MVs by dedicated small-particle flow cytometry

**Figure 1:**
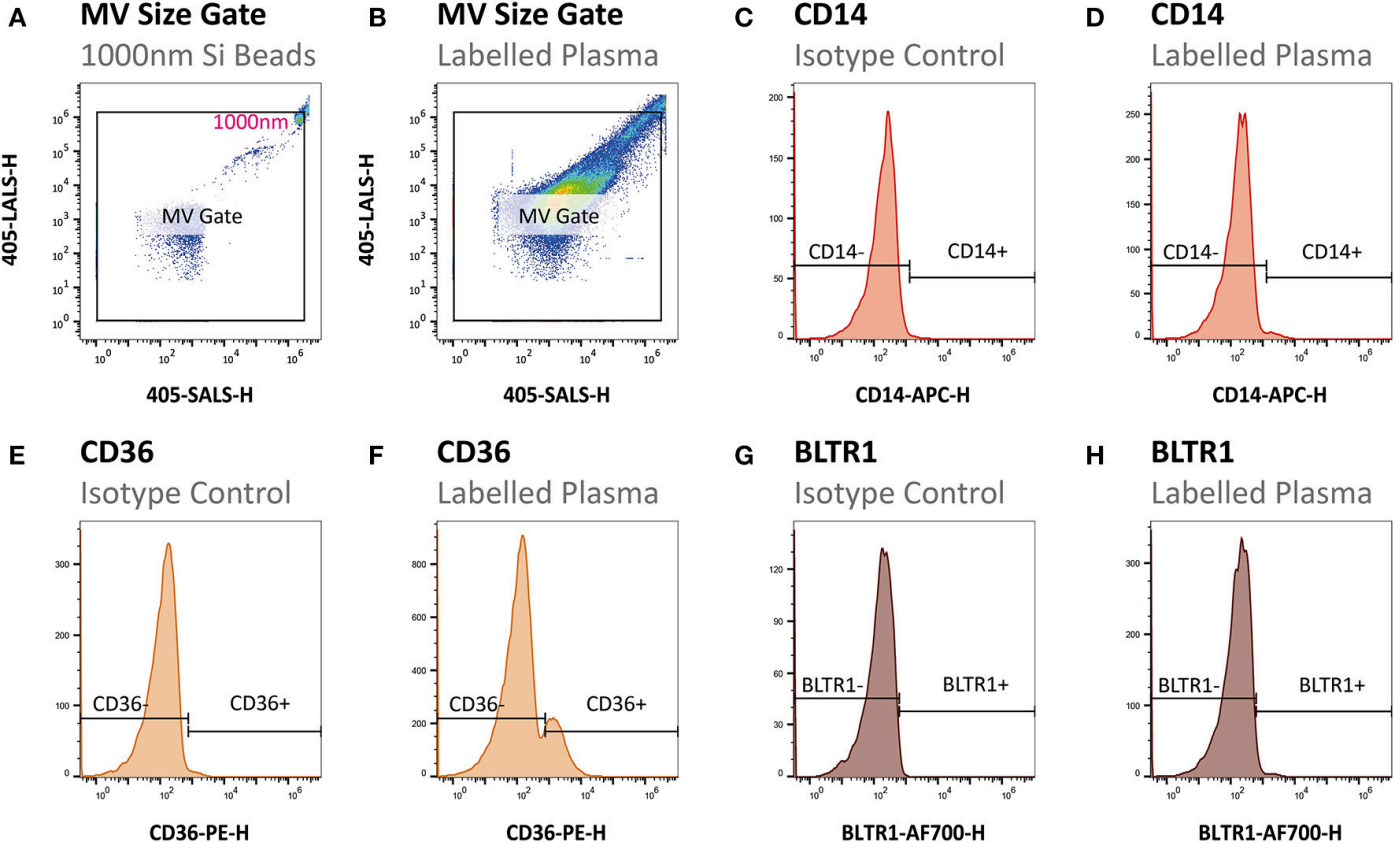
Flow cytometric gating strategy of MVs. **(A)** The MV gate was determined using size-calibrated green fluorescent silica beads with the size of 1,000 nm and **(B)** applied to all samples. **(C,D)** Next, MMVs (CD14^+^) were gated by log-scaled APC fluorescence peak intensity (APC-H) at the 99^th^ percentile of isotypes. Finally, CD36^+^ MVs **(E,F)** and BLTR1^+^ MVs **(G,H)** were gated by log-scaled PE fluorescence peak intensity (PE-H) at the 99th percentile of isotypes and log scaled AF700 fluorescence peak intensity (AF700-H) at the 99th percentile of isotypes, respectively.

Samples were analyzed using an A-60 Micro-PLUS flow cytometer (Apogee Flow Systems, Hertfordshire, United Kingdom). The sample flow rate was 0.75 μL/min and the time of acquisition was 600 s for all samples. In order to exclude excessive background noise a combined threshold on light scatter and green fluorescence was set above background using PBS and an un-labeled plasma sample, respectively. Statistics from flow cytometry data was extracted and gating was conducted as illustrated in Figure 1 in FlowJo version 10.4 (FlowJo LLC, Oregon, USA). First, the upper limit of the MV gate was determined using size-calibrated green fluorescent silica beads with the size of 1,000 nm and then applied to all samples (Figures 1A,B). Second, MVs were defined as events within the established size gate expressing PS indicated by binding of lactadherin-FITC. Third, MVs originating from monocytes were defined as CD14 positive events gated by log-scaled APC fluorescence peak intensity (APC-H) at the 99th percentile of isotypes (Figures 1C,D). Fourth, the MVs expressing CD36 and BLTR1 were defined as CD36 and BLTR1 positive events on MVs and on the MMV subfraction gated by log-scaled PE fluorescence peak intensity (PE-H) at the 99th percentile of isotypes and log-scaled AF700 fluorescence peak intensity (AF700-H) at the 99th percentile of isotypes, respectively (Figures 1E–H).

### Statistics

Statistical analyses were carried out using IBM SPSS Statistics version 24.0 (SPSS, Armonk, NY) and GraphPad Prism 6.0 (GraphPad Software, La Jolla, California, USA). Categorical variables were compared with the Chi^2^ test. Continuous variables were tested for normality and homoscedasticity by Shapiro-Wilk's W test and Levene's test for equal variance, respectively. Continuous variables were depicted as boxplots with median and 25–75th percentiles (box) and 95% confidence intervals (whiskers). Normally distributed data with equal variances between groups were compared using Student's *t*-test. Heteroscedastic or non-normally distributed variables were compared using the Mann-Whitney *U-*test. Statistical correlations were analyzed in participants with femoral atherosclerosis using Spearman's ranked correlation analysis. All *p*-values are two-sided, and statistical significance was defined as *p* < 0.05.

## Results

### Characteristics of the study population

The mean age for the 48 atherosclerotic patients and 24 controls was 70.9 ± 10 and 47.1 ± 9.9 years, respectively. Both sexes were represented with 50% males in the patient cohort and 60% males in the control group. The median BMI was 25.2 ± 4.8 kg/m^2^ for patients and 23.7 ± 3.2 kg/m^2^ for controls. The level of hsCRP was almost two-fold higher in the patient cohort compared to the control group (*p* < 0.015). Additional parameters are listed in Table 1.

**Table 1:** Baseline characteristics of study population.

	**Atherosclerotic patients**	**Healthy controls**	***p*-value**
	**(*****n*** = **48)**	**(*****n*** = **24)**	
**AGE AND GENDER**			
Age *(years)*	70.9 ± 10	47.1 ± 9.9	<0.001^*^
Sex *(male/female)*	24/24	9/15	0.452
Body mass index (kg/m^2^)	25.2 ± 4.8	23.7 ± 3.2	0.122
Current smokers (%)	35	17	0.168
**MEDICAL HISTORY AND MEDICATIONS**			
Cardiovascular disease^†^ (%)	58	–	–
Diabetes mellitus (%)	24	–	–
Aspirin treatment (%)	98	–	–
Anti-hypertensive medication (%)	78	–	–
Statin treatment (%)	96	–	–
NSAID use^‡^ (%)	7	–	–
EPA + DHA intake (g/day)	0.67 (0.32; 1.21)	–	–
**LABORATORY EXAMINATION**			
Total cholesterol (mmol/l)	3.8 (3.4; 4.4)	5.05 (4.58; 5.45)	<0.001^*^
LDL cholesterol (mmol/l)	1.75 (1.5; 2.1)	2.9 (2.48; 3.30)	<0.001^*^
HDL cholesterol (mmol/l)	1.4 ± 0.42	1.71 ± 0.39	0.003^*^
Plasma triglyceride (mmol/l)	1.3 (1; 1.93)	1.0 (0.8; 1.2)	0.02^*^
Plasma glucose (mmol/l)	5.9 (5.5; 6.5)	5.35 (5.08; 5.8)	<0.001^*^
Plasma hsCRP^§^ (mg/l)	1.8 (0.83; 4.67)	0.94 (0.67; 1.45)	0.014^*^
LTB4 in granulocytes (ng/10^7^ cells)	203.0 ± 41.1	–	–
LTB4 in plasma (pg/ml)	133.0 ± 108.2	–	–
LTB4 in plaques (pg/mg)	5.4 ± 4.9	–	–
AA in granulocytes (% of total fatty acids)	13.1 ± 1.2	–	–
AA in plasma (% of total fatty acids)	10.5 ± 2.3	–	–
AA in plaques (% of total fatty acids)	6.4 ± 2.2	–	–
EPA in granulocytes (% of total fatty acids)	0.7 ± 0.3	–	–
EPA in plasma (% of total fatty acids)	2.1 ± 1.0	–	–
EPA in plaques (% of total fatty acids)	1.0 ± 0.4	–	–

*Data are depicted as mean ± SD or median (Q_25%_; Q_75%_)*.

* *Statistically significant difference between patients and healthy controls*.

† *Any diagnosis of stable angina, unstable angina, MI, ischemic stroke, transitory ischemic attack or any procedure of percutaneous coronary intervention or coronary artery bypass grafting*.

‡ *Chronic use of non-steroidal anti-inflammatory drugs (NSAID)*.

§ *Patients with hsCRP > 10 mmol/l (n = 9) were omitted since these values suggest acute rather than low-grade inflammation. Bold values represents statistical significance*.

### Patients had lower levels of circulating BLTR1 expressing MV phenotypes compared to controls

**Figure 2:**
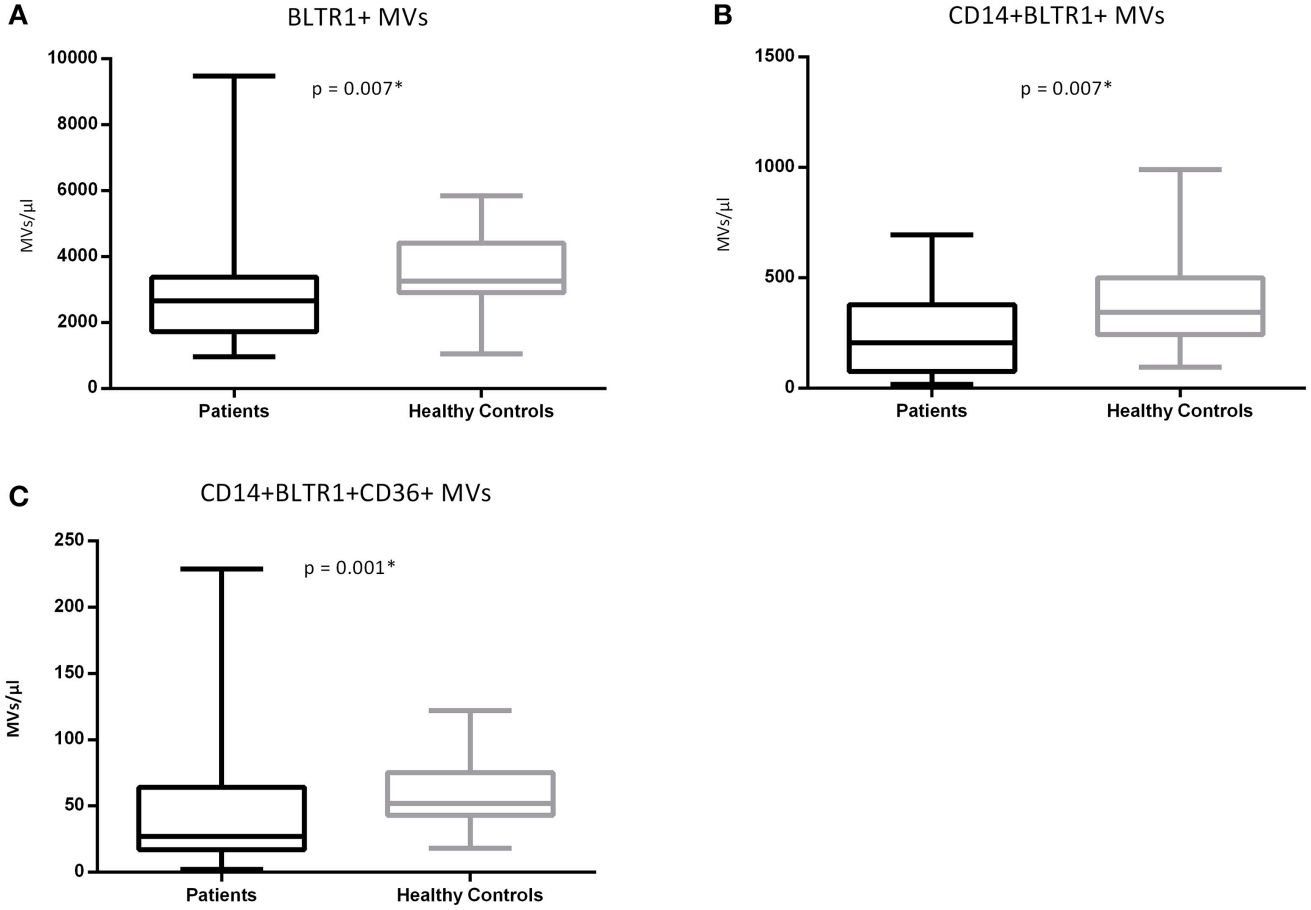
**(A–C)** BLTR1^+^ MVs in atherosclerotic patients (*n* = 48, black) and healthy controls (*n* = 24, gray). Data are represented in MVs/μl and are depicted as boxplots with whiskers as 95% confidence intervals.

No significant differences in total MV concentration (*p* = 0.246), CD14^+^ MVs (*p* = 0.277), CD36^+^ MVs (*p* = 0.656), and CD14^+^CD36^+^ MVs (*p* = 0.115) between groups were observed. Patients with femoral atherosclerosis had lower levels of BLTR1^+^ MVs (*p* = 0.007), CD14^+^BLTR1^+^ MVs (*p* = 0.007), and CD14^+^BLTR1^+^CD36^+^ MVs (*p* = 0.001) compared to controls (Figure 2). Age was not significantly correlated with any of the investigated MV phenotypes in neither the patient cohort (*rho* from −0.05 to −0.22, *p* from 0.13 to 0.74) nor the control group (*rho* from −0.24 to 0.33, *p* from 0.11 to 0.88).

### MMV sub-phenotypes correlated inversely with AA content in patients

**Figure 3:**
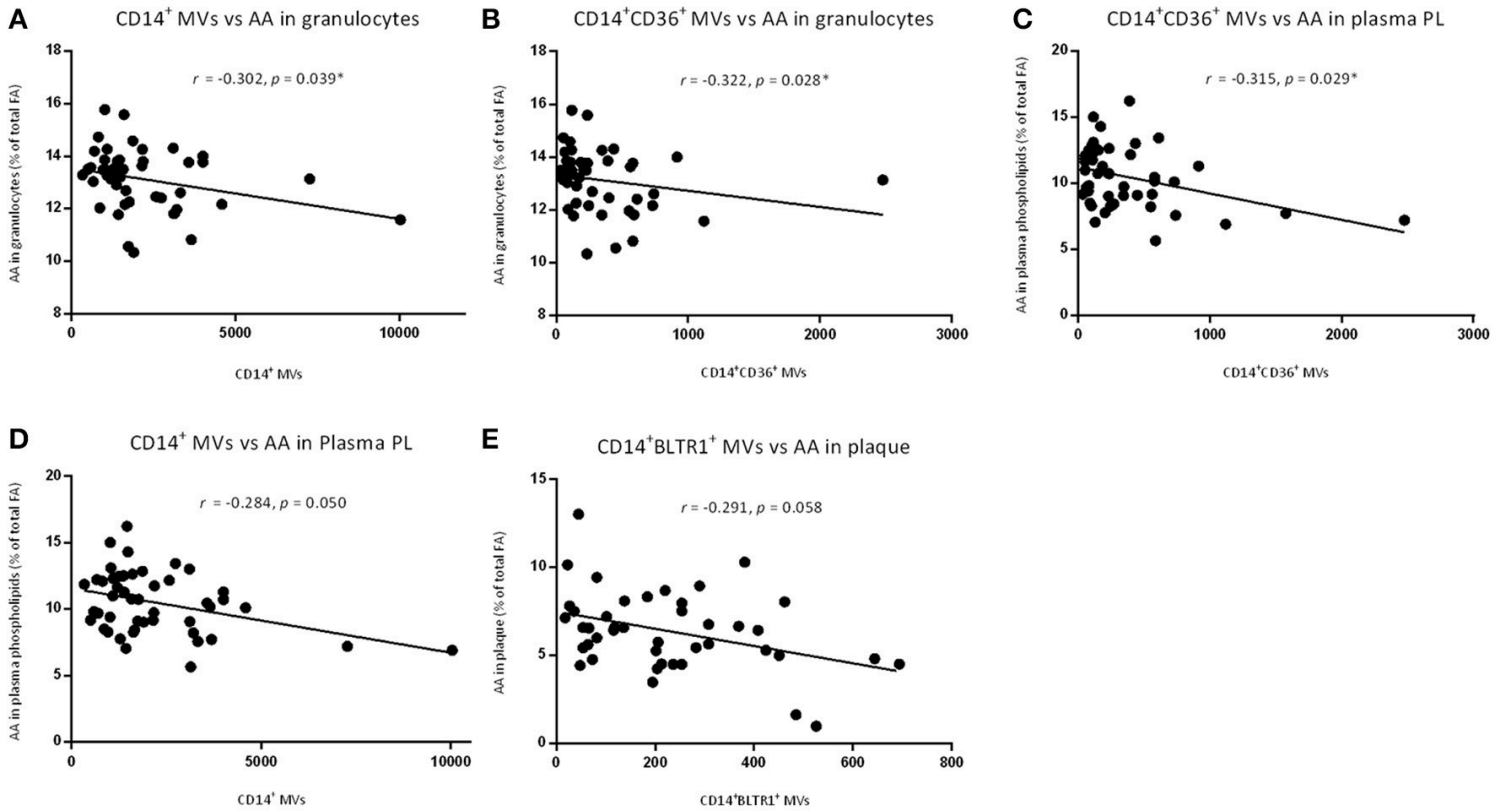
Correlations of different MV phenotypes and arachidonic acid (AA) content. Correlations of **(A)** CD14^+^ MVs and AA in granulocytes, **(B)** CD14^+^CD36^+^ MVs and AA in granulocytes, and **(C)** CD14^+^CD36^+^ MVs and AA in plasma phospholipids. Nearly correlations of **(D)** CD14^+^ MVs and AA in plasma phospholipids and **(E)** CD14^+^BLTR1^+^ MVs and AA in plaques. AA content in different compartments are measured in % of total fatty acids.

Levels of CD14^+^ MVs and CD14^+^CD36^+^ MVs correlated inversely with AA in granulocytes (*r* = −0.302, *p* = 0.039, and *r* = −0.322, *p* = 0.028, respectively; Figures 3A,B). CD14^+^CD36^+^ MVs further correlated inversely with AA in plasma phospholipids (*r* = −0.315, *p* = 0.029; Figure 3C). The level of CD14^+^ MVs and CD14^+^BLTR1^+^ MVs tended to correlate inversely with AA in plasma phospholipids and AA in plaques, respectively (*r* = −0.284, *p* = 0.050 and *r* = −0.291, *p* = 0.058; Figures 3D,E). No correlations were identified between MV level and content of EPA in plaques, granulocytes, and plasma phospholipids as well as MV level and content of LTB4 in plaques, granulocytes, and plasma phospholipids.

### MMVs expressing CD36 correlated with triglyceride levels in both patients and healthy controls

CD14^+^ MVs and CD14^+^CD36^+^ MVs correlated with plasma triglyceride in both patients (*r* = 0.33, *p* = 0.022 and *r* = 0.34, *p* = 0.019, respectively) and controls (*r* = 0.38, *p* = 0.063 and *r* = 0.46, *p* = 0.022, respectively). Furthermore, BLTR1^+^ MVs correlated with plasma triglyceride, and tended to correlate inversely with LDL (*r* = 0.31, *p* = 0.028 and *r* = −0.27, *p* = 0.062, respectively).

### Effect of elevated hsCRP on MV phenotypes in patients

To investigate a possible effect of inflammation, MV phenotypes from patients with hsCRP >10 mmol/l (*N* = 9) were compared to patients with hsCRP ≤10 mmol/l (*N* = 39). No significant differences were found in total MVs (*p* = 0.45), CD14^+^ MVs (*p* = 0.98), CD36^+^ MVs (*p* = 0.21), BLTR1^+^ MVs (*p* = 0.76), CD14^+^CD36^+^ MVs (*p* = 0.97), CD14^+^BLTR1^+^ MVs (*p* = 0.19), and CD14^+^CD36^+^BLTR1^+^ MVs (*p* = 0.14).

## Discussion

This study was conducted to investigate the potential association of circulating CD36^+^ and BLTR1^+^ MMVs with femoral atherosclerosis on the one hand, and AA and EPA contents in atherosclerotic plaques, plasma, and granulocytes on the other. We hypothesized that patients with femoral atherosclerosis would have higher levels of MMVs, in particular those expressing CD36 and BLTR1, compared to healthy controls. We further hypothesized that patients with high content of AA would have associated elevated levels of these MMVs.

Unexpectedly, in the present study we found that the concentration of total MVs, CD14^+^ MVs, CD36^+^ MVs, and CD14^+^CD36^+^ MVs were not increased in the patient cohort compared to healthy controls. Atherosclerosis is a chronic inflammatory disease of the vessel wall and several studies have shown that inflammation leads to increased leukocyte-derived MV release (12). Further, circulating leukocyte-derived MV levels might predict subclinical atherosclerosis in asymptomatic individuals (12), and it was thus unexpected that the patients did not have higher levels of CD14^+^ MVs, and that these MV phenotypes were unaffected by inflammation. In addition, we have previously demonstrated that patients with familial hypercholesterolemia and thereby increased risk of atherosclerosis, had higher levels of both CD14^+^ MVs and CD14^+^CD36^+^ MVs compared to healthy controls (13). Furthermore, other investigators demonstrated an association between cardiovascular disease and increased CD14^+^ MVs and total MV concentration (14, 15). One possible explanation for our observations is that the patients in this study were receiving pharmaceutical treatment including statins, which may potentially reduce the MV concentration (16). Moreover, age has been proposed to affect vesicle concentration with decreasing number in advancing age (17). Since we were unable to demonstrate any significant association of MV phenotypes with age, it is unlikely that the reduced level of BLTR1^+^ MVs in the atherosclerotic patient cohort can be attributed to increased age compared to the healthy controls.

The AA-derived LTB4 has been linked to atherosclerosis mainly through BLTR1 (8, 18) and a reduced concentration of BLTR1^+^ MVs in the atherosclerotic patients was thus unexpected. However, a number of studies (19, 20) have demonstrated a chemotactic deactivation of leukocytes to LTB4, which is attributed a diminished expression of BLTR1 by converting the high affinity BLTR1 to the low affinity BLTR2 and/or internalization of BLTR1 when exposed to LTB4. Chou et al. (21) also documented a decreased BLTR1 expression after leukocytes were exposed to LTB4 in infiltrated arthritic joints. Atherosclerotic patients tend to have higher levels of LTB4 than healthy controls (22) that could lead to a down-regulation of the receptor and thus lower levels of BLTR1^+^ MVs in atherosclerotic patients, as found in the present study. In relation to our findings of decreased CD14^+^BLTR1^+^ MVs, Tangirala et al. (23) reported that differentiation from monocytes to macrophages by IFN-γ stimulation led to a rapid loss in chemotactic response and further resulted in downregulation of their chemotactic surface receptor by 40%. Pettersson et al. (24) also demonstrated that pro-inflammatory cytokines including TNF-α, IFN-γ, and LPS down-regulated BLTR1 surface expression and mRNA expression in human monocytes opposed to the anti-inflammatory cytokine IL-10 that up-regulated BLTR1 expression. In addition, they showed that the phenotypic pro-inflammatory CD14^+^CD16^+^ monocytes had lower expression of BLTR1 and CCR2 compared to the classical CD14^++^CD16^−^ monocytes expressing high levels of the receptors. In our study, hsCRP levels were significantly higher in patients compared to healthy controls. Moreover, there was a tendency toward decreased CD14^+^CD36^+^BLTR1^+^ MVs, although not significant. Therefore, it is likely that the chronic low-grade inflammatory condition with abundance of the pro-inflammatory monocyte phenotype in patients with femoral atherosclerosis together with higher levels of LTB4 may further contribute to a down-regulation of the BLTR1 and thus be in line with our findings of reduced BLTR1^+^ MVs and CD14^+^ MVs.

In patients, we observed inverse correlations between levels of CD14^+^ MVs and CD14^+^CD36^+^ MVs and AA in different compartments. As previously described, CD36 on monocytes enhances the atherosclerotic progression (4, 25–28) and is further upregulated by LTB4 (11). Therefore, we would have expected that the content of AA, the precursor for LTB4, was positively correlated with the level of CD14^+^ MVs and CD14^+^CD36^+^ MVs but surprisingly this was not the case. It should be noted that the major risk factor for femoral atherosclerosis is not AA content but cigarette smoke (29), which might explain the unexpected results. However, cigarette smoke is linked to chronic inflammation and is found to increase the CD14^+^CD36^+^ cell population (30) thus most probably increasing the level of CD14^+^CD36^+^ MVs. Importantly, the patients in the current study were treated with aspirin (98%) and statins (96%) which might lower the levels of CD36^+^ MVs and CD14^+^ MVs as studies have demonstrated lower levels of sCD36 when treated with acetylsalicylic acid (31) and several MV phenotypes, including CD14^+^ MVs, when treated with statins (16). This may have affected our results opposing what was expected.

Interestingly, we found that the level of CD14^+^ MVs and CD14^+^CD36^+^ MVs correlated positively with triglyceride in both patients and healthy controls, in accordance with the function of CD36 as a fatty acid transporter. In previous studies, we demonstrated similar associations in type 2 diabetic males (32) and in severely obese individuals (unpublished results). The lack of correlations between cholesterol fractions and MMV may be secondary to statin treatment and the resulting reduction of total cholesterol and LDL as the primary effect, whilst there is only minor impact on triglycerides (33).

The current study has significant strength in several ways despite its surprising results. To the best of our knowledge, this study is the first to investigate BLTR1^+^ MVs and further, the correlation between BLTR1^+^ MVs and the content of EPA and AA in atherosclerotic patients. Thus, hypotheses in this explorative study were based on clinical associations and cell studies, which makes it more difficult to generate precise hypotheses. Methodologically, flow cytometry is a high-throughput method that holds the ability to simultaneously characterize single MVs in regards to quantification, size distribution, and expression of multiple surface markers, which makes flow cytometry a preferred method in the study of MVs. Nonetheless, traditional flow cytometers lack the ability to detect the smallest MVs and have a lower cut-off limit of between 200 and 500 nm. We have developed a novel method for detection of rare and small MV phenotypes, including BLTR1^+^ MVs, utilizing a dedicated small-particle flow cytometer (spFCM) with high sensitivity together with a novel data acquisition strategy allowing detection of even very small and rare MVs. In addition, this is the first study, using a spFCM that measures MVs directly in plasma without purification, thus providing the most authentic information of the mechanisms linked to development and progression of the disease.

A number of limitations in this study should also be addressed. First, the match between patients and controls is not optimal. The big difference in age could have affected the level of several MV phenotypes, which might explain the missing findings of increased MV levels in patients that we would have expected. However, it would be very difficult to find healthy controls with the age of 70 years without plaque or other age-related diseases. In addition, we found no significant correlation between age and any of the investigated MV phenotypes. Further, the patients in this study were receiving pharmaceutical treatment including aspirin and statins. This has been shown to reduce the level of several MV phenotypes including CD14^+^ and CD36^+^ MVs. Although treatment of the patients is a limitation of the study, atherosclerotic patients with such advanced disease that render them suitable for thrombendarterectomy, and thereby plaque characterization, are always receiving pharmacological treatment hence this could most likely not have been avoided. Moreover, we only have measurements of circulating LTB4, AA, and EPA in patients and not in controls, which would have been desirable. Lastly, MVs analyzed encompasses only monocyte-derived MVs and not MVs derived from other cell types. As atherosclerosis is a multifaceted disease, it would have been desirable further to analyse MVs from platelets and especially endothelial cells among others.

## Conclusion

In summary, the present study demonstrated no differences in the levels of total MVs, CD14^+^ MVs, CD36^+^ MVs, and CD14^+^CD36^+^ MVs between patients and controls. Furthermore, BLTR1^+^ MV phenotypes were significantly lower in atherosclerotic patients compared to healthy controls and CD14^+^ MVs and CD14^+^CD36^+^ MVs correlated inversely with AA in granulocytes, and CD14^+^CD36^+^ with AA in plasma phospholipids. These unexpected findings may be a result of the confounding effects of pharmacologic treatment of the atherosclerotic group. We did find a positive correlation between the level of CD14^+^ MVs and CD14^+^CD36^+^ MVs with triglyceride in both patients and healthy controls, which is in accordance with the function of CD36 as a fatty acid transporter. Since this is the first study of its kind, the results are explorative and only indicative, and additional studies are necessary to contribute to a more detailed understanding of the relationship between MVs and the PUFA content in patients with femoral atherosclerosis, and the potential of MVs as markers of atherosclerosis pathophysiological mechanisms.

## Author contributions

MS, JB, and AH designed the study. MSN, ES, and MHN collected samples and data. MS and JB performed the experiments. MS analyzed data and was the major contributor in writing the manuscript supervised by AH. All authors approved the final version of the manuscript.

### Conflict of interest statement

AH has a patent WO2005/116644 issued. AH is the inventor of a patent application on sCD36 as a biomarker of atherosclerosis risk. The patent IP rights are owned by the Idea's Clinic of Aalborg University Hospital. The remaining authors declare that the research was conducted in the absence of any commercial or financial relationships that could be construed as a potential conflict of interest.
